# We will make you like our research: The development of a susceptibility-to-persuasion scale

**DOI:** 10.1371/journal.pone.0194119

**Published:** 2018-03-15

**Authors:** David Modic, Ross Anderson, Jussi Palomäki

**Affiliations:** 1 University of Cambridge Computer Laboratory, Cambridge, England; 2 Cognitive Science, Department of Digital Humanities, University of Helsinki, Helsinki, Finland; Universidad Nacional de Mar del Plata, ARGENTINA

## Abstract

Psychological and other persuasive mechanisms across diverse contexts are well researched, with many studies of the effectiveness of specific persuasive techniques on distinct types of human behaviour. In the present paper, our specific interest lies in the development of a generalized modular psychometric tool to measure individuals’ susceptibility to persuasion. The scale is constructed using items from previously developed and validated particulate scales established in the domains of social psychology and behavioural economics. In the first study we establish the Susceptibility to Persuasion–II (StP-II) scale, containing 54 items, 10 subscales and further 6 sub-sub scales. In Study 2 we establish the scale’s construct validity and reconfirm its reliability. We present a valid and reliable modular psychometric tool that measures general susceptibility to persuasive techniques. Since its inception, we have successfully implemented the StP-II scale to measure susceptibility to persuasion of IT security officers, the role of psychology of persuasion in cybercrime victims and general persuadability levels of Facebook users; these manuscripts are in preparation. We argue that the StP-II scale shows promise in measuring individual differences in susceptibility to persuasion, and is applicable across diverse contexts such as Internet security and cybercrime.

## Introduction

Many specific psychological mechanisms of persuasion have been well researched in the past, notably by Cialdini [1]; and Knowles and Linn [2]. The predominant approach is to focus on one mechanism and explore its effect in a specific context. Several researchers have noted that this presents only a partial view of persuasive mechanisms (cf. [2]). In the present paper we create, test and validate a unifying scale of Susceptibility to Persuasion (StP-II), which brings together social and consumer psychological mechanisms that have proven to be applicable across different contexts.

The primary purpose of StP-II is to measure factors that play a role in compliance with fraudulent offers (i.e. scam compliance). Other applicable areas may include consumer and marketing psychology and behavioural economics. In preceding research Fischer, Lea [3] compiled a report for the UK Office of Fair Trading (OFT) on factors that have an impact on scam compliance, such as consumers’ need for uniqueness and sensation seeking. They have also shown that scam compliance can be likened to an error in judgment: vulnerability to persuasion is not directly linked to intelligence, but to mechanisms that momentarily suspend the rational decision-making processes [4].

As an extension of the OFT report, Modic and Lea [5] investigated the psychological factors that cause such momentary lapses of judgement. They confirmed that a number of mechanisms, such as social influence and lack of self-control, play a role in scam compliance. In addition to testing the mechanisms established by Fischer, Lea [4], Modic and Lea [6] explored additional personality traits that affect scam compliance. Agreeableness and introversion were shown to play a role as well as lack of premeditation (a component of Impulsivity; [7]). These findings were in line with other research in this context, such as from Duffield and Grabosky [8]; and Buchanan and Whitty [9].

An initial version of the scale of Susceptibility to Persuasion (StP) was then developed and tested on the responses of fraud victims by Modic and Lea [5]. While StP adequately measured scam compliance, with high factor loadings on various subscales and good reliability, several concerns arose. The initial version of StP was developed from scratch and did not incorporate previous scale development efforts. Furthermore, the studies’ ecological validity was not established beyond doubt. In addition, StP only reliably measured four constructs related to susceptibility to persuasion (Influence of Authority, Social Influence, Need for Consistency, and Self-control; [6]). While this was a good starting point for StP-II, we derived additional subscales based on indications from Modic’s PhD research [10], which in turn derived viable mechanisms from Fischer, Lea [3], Knowles and Linn [2], and other sources mentioned in this manuscript. For a more detailed theoretical rationale on the foundation of the original susceptibility to persuasion–scale, see [10].

This paper significantly extends the initial version of the Susceptibility to Persuasion Scale. Various theoretical underpinnings (e.g. [2, 9, 11–13] and empirical results, e.g. [4, 5, 14] have given us the basis for creation, factorisation, and validation of a new scale measuring susceptibility to persuasion (StP-II).

### Scam compliance and susceptibility to persuasion

While there is a commonly held belief that an individual can either be a victim of a scam or not, there is a growing amount of evidence that this is perhaps too simplistic. Fischer, Lea [4] hint that the process of becoming a victim of fraud (i.e. scam compliance) is more intricate than simply falling for fraud or not. Shadel [15] qualitatively postulates that falling for fraud is a process consisting of four stages (the front, the drive, the close and the load), roughly corresponding to getting the potential victim excited about the possibility of large gains, developing the relationship, taking the money, and finally repeating the scam on the same individual. Modic and Lea [6] and Modic and Anderson [16] postulate three stages of scam compliance, that is plausibility, interaction with (or responding to) fraudsters, and losing utility to fraud. Simply put, a potential victim will first decide whether a specific fraudulent scheme is believable (or plausible), then interacts with the scammer and finally loses utility to them. It would make sense that different psychological mechanisms influence different stages of scam compliance, as has been demonstrated by various researchers [6, 8, 17, 18]. Moreover, it has been demonstrated repeatedly that constructs related to impulsivity and lack of self-control, either as a trait [19] or as a cognitive state [20] will play an important part in increasing scam compliance [2, 4–6, 10, 19, 21]. Therefore, any scale that measures susceptibility to persuasion should include related constructs, such as sensation seeking [22, 23], impulsivity subconstructs such as premeditation [24], and self-control itself [25].

Scam compliance could be construed as a subset of consumer behaviour, that is scams can be seen as an illegitimate marketing offer [3–5]; therefore marketing mechanisms such as the need for uniqueness [26] and the attitude towards marketing offers [27] would have to be included. In the same vein, persuasive techniques that have proven to be efficient in other fields should also be salient in scam compliance. Therefore, a number of persuasive mechanisms developed by Cialdini [1] will need to be included in a new scale.

Susceptibility to persuasion (i.e. *persuadability*) is a phenomenon of the subject who is persuaded, but is influenced by the plausibility of the story of which they are persuaded. Vaughn, Hesse [28] have shown that when individuals find a narrative plausible, they are more likely to be persuaded to invest in it. Pornpitakpan [29] has shown that, in advertising and other contexts, a credible source increases plausibility of a message, which has a significant impact on persuadability. In Internet fraud, individuals who found scams plausible were more likely to comply with social influence and influence of authority [5]. In the same vein, it is likely that a person who finds a (legal or illegal) marketing offer believable will respond to it, regardless of cultural context [30]. And once they have responded to it, they are likely to financially invest in it, since they already invested time and energy, and the sunk cost fallacy [31] applies. In the present article, we will establish the construct validity of StP-II through comparison with self-reported scam compliance. We expect to find that individuals who are susceptible to persuasion will give credence to fraudulent marketing offers which more cautious people would not find convincing. These susceptible individuals will then also likely respond to the scams they found credible as well as lose utility to them.

## Method 1

### Participants

Our respondents for this study were recruited via Amazon Mechanical Turk (mTurk) between November 2013 and February 2014. There were five sittings, where we collected between 100 and 200 unique responses each time. Each respondent was paid US $0.35 for participating and additional US $0.25 if they responded to more than 90% of the scale items. In three out of five sittings only participants based in the US could answer the questionnaire, while the other two sittings were open to the world. In total 998 mTurkers responded. Data files were then screened for duplicates and empty responses, leaving us with 779 unique respondents who answered most of the scale items and did not fail the demand characteristics check; that is, the remaining respondents did not report understanding the purpose of the experiment beforehand.

In our sample, age (a categorical variable) was distributed in a curve that resembled normal distribution with a peak in the 31–40 range (n = 203; 26%). Gender was self-reported as 327 (43%) female and 433 (57%) male (19 respondents refused to answer this question). Most respondents reported their level of education as BA/BSc or similar (n = 398; 52%). Most respondents were born in India (n = 375; 48%) or United States of America (n = 359; 46%). Country of residence was the same as country of birth for 739 respondents (97%). Out of the remaining respondents 60% listed US as their country of residence (living there for 5 years or more). More demographics are given in the Results Section. Most of the respondents reported being employed in the private sector (n = 319; 41%), followed by self-employment (n = 99, 13%), employment in the public sector (n = 99; 13%). All but 21 (3%) of the remaining participants who disclosed their occupational status did not receive regular pay for their current work. They were unemployed/did casual work (n = 62; 8%), were caring full-time for a family member (n = 61; 8%), were students (n = 83, 11%), were retired (n = 15, 2%) or not working due to disability (n = 19, 2%). A single additional respondent reported running their own business that has other employees. Respondents reported their IT knowledge to be better than average, with 76% scoring themselves more than 3 on a five-point scale (mean = 4.13, SD = 0.989).

### Scale development

The initial version of the StP-II scale consisted of 138 items in 9 subscales, with 5 of the initial subscales consisting of further two or three subscales. The salient factors and their corresponding subscales were picked based on pre-existing empirical and theoretical research into scam compliance. They are as follows.

Social influence (SI) was shown to be an important predictor of scam compliance by Modic and Lea [5] in the initial StP scale. This confirms the findings of Weeks, Ardevol-Abreu [32] who have shown that social influence is salient in online contexts such as social media. While there is some research showing the existence of context effects when it comes to virtual social constructs [33], there is abundant evidence that social influence plays a role in virtual contexts [34–37]. The issue is therefore not whether Social Influence is salient in the context of online persuasion, but rather to what extent.

While Social Influence is a complex construct, we were constrained by the nature of the psychometric tool we were using. While, for example, the concept of non-dynamic social influence is now considered to be somewhat dated, and seen as a mechanism contributing to social identity theory [34, 38], it is difficult to include a dynamic measurement into a static psychometric tool. Therefore, we settled on a static measure of SI, which would still give us an indication of how likely an individual is to be influenced by social pressure. The SI items used in StP-II have been refined by Batra, Homer and Kahle [39]. The initial scale on which the current one is based on (susceptibility to interpersonal influence, SII; 12 items) was developed by Bearden, Netemeyer and Teel [40] and consists of two subscales that measure normative (8 items); and informative (4 items) social influence.

Need for Cognition–the concept of individual’s need to ascribe meaning and purpose to events was first postulated by Cohen, Stotland [41] and later developed by Cacioppo and Petty [42]. The StP-II subscale items were collected from the NCS scale (18 items) refined by Cacioppo, Petty [43]. Note that the need for cognition has been tied to self-consciousness [44]; and sensation seeking [45] which both have a tangential bearing on scam compliance [5].

Need for Consistency was one of the initial factors influencing compliance postulated by Fischer, Lea [3]. Consistency emerged as a significant predictor in the original StP scale [5] and has been flagged by Cialdini [1] as one of the six basic tendencies that generate a positive response to persuasion. The StP-II subscale items were taken from the Preference for Consistency (PFC-B scale; 8 items) developed and validated by Cialdini, Trost [46].

A scam is in many ways similar to a marketing offer [4] although the end result of a scam is illegal or exploitative. In advertising in general, persuasive techniques are employed as discussed for example by Thakor and Goneau-Lessard [47] and by Obermiller and Spangenberg [48]. Much recent research has focused on scepticism towards health-related advertising while general Attitudes Towards Advertising have been somewhat neglected. Gaski and Etzel [27] conducted a large survey on consumer attitudes that includes the Attitude Towards Advertising (ATA) scale that was initially developed by Bauer, Greyser [49] and further modified by Andrews [50]. ATA has seven items in two subscales measuring Social (3 items) and Economic (4) dimensions. Earlier findings (e.g. [4]) suggest that attitudes towards advertising should play a role in scam compliance. That is, people with a more positive attitude towards advertising will be more likely to go along with a marketing offer, even though it might be a scam.

Sensation Seeking has been shown to influence impulsive behaviour [24], which in turn has an impact on compliance [6]. In StP-II construction, we faced several options–we could have used a specific subscale from UPPS-IBS [7] or one of the standalone inventories. The Sensation-Seeking Scale (SSS-V; [51]) is well established, continuously adapted, and refined (e.g. [22]). However, the format of SSS-V is not suitable for StP-II (it is a forced choice, two-outcome questionnaire, since all the other subscales in this instrument are measured with Likert-type variables). Another established sensation-seeking scale is the Arnett Inventory of Sensation Seeking (AISS; [23]). The AISS (20 items) refined by Haynes, Miles [52] consists of two subscales measuring novelty (10 items); and intensity (10 items). Note that Haynes, Miles [52] had to remove some AISS items due to low factor loadings. We encountered similar issues in our analysis.

Self-control is an important predictor of a diverse set of behaviours, for example victimization in general [25, 53, 54] and fraud specifically [17]. The ability to exert self-control reduces the effect of demographic factors such as gender and income on falling victim to fraud [19, 21]. Individuals with low self-control have difficulties controlling their emotions, leaving them vulnerable to errors in judgment [25] that lead to less than optimal decisions when responding to scams [55]. For StP-II we used the 13 items found in the Brief Scale of Self–Control (BSCS) as listed by Holtfreter, Reisig [19] and originally developed by Tangney, Baumeister [25].

Lack of Premeditation; or Consideration of Future Consequences is an intrinsic part of impulsivity [7] and a significant predictor of scam compliance [6]. For StP-II, we used the 12-item CFC scale developed by Strathman, Gleicher [56] and further confirmed by recent research [57, 58].

Need for Uniqueness and Avoidance of Similarity drives certain aspects of consumer behaviour. Research has shown consumers to be likely to respond positively to marketing offers when they believed that the goods on offer to be unique or scarce [59–61]. In scam research, Langenderfer and Shimp [55] have shown that many scams utilize that phenomenon to pronounced effect. The salient subscale in StP-II was constructed from the 16 item short form of the Consumer Need for Uniqueness scale (CNFU-S), with four subscales measuring Creative Choice (4 items), Unpopular Choice (4 items), Avoidance of Similarity (4 items) and Unique Consumption behaviour (4 items) refined by Ruvio, Shoham [26] from the original CNFU (31 items) introduced by Tian, Bearden [62].

Risk Preferences Across Contextual Domains as postulated by Weber, Blais [63]. Risk preferences have been shown to play a strong role in decision making in general [64]. Recent research suggests that attitudes towards risky choices vary according to the context [65, 66]. It would be reasonable to infer that risk preferences when it comes to fraud would be similar to other domains with similar characteristics (e.g. finance and ethics). In StP-II we used the full DOSPERT-R scale established by Weber, Blais [63], but pruned it down to specific reliable and salient domains in later analysis.

### Design

To control for order effects the items in particulate scales were randomised. The survey was delivered online. All participants answered the exploratory and demographic questions at the start of the survey. The study was approved by the University of Cambridge ethics committee. Confirmatory factor analysis was conducted using R 3.4.0 package with the lavaan extension.

### Procedure

The survey was delivered online, and consisted of five sequential parts:

1. Introduction to the experiment, with a brief explanation of the structure and our reasoning for using it; assurance of anonymity; and a request for permission to use the data in the analysis.
2. Demographic items (gender, age, familiarity with computers, educational level, country of birth, country of residence, occupational status)
3. 9 Scales: SII, NCS, PFC-B, AISS, ATA, BSCS, CFC, CNFU-S, DOSPERT-R
4. Demand characteristics questions, general questions.
5. Debriefing.

## Results 1

Initial response data (n = 779) were separated into two groups using random sampling as, for example, suggested by Pohlmann [67]. The Main group contained 500 responses, while the Holdout group contained 279 responses. The StP-II scale initially consisted of 136 items. The standardized Cronbach alpha score for the whole scale on the full sample was .958 (n = 779).

### Reliability testing

Prior to exploratory factor analysis each, of the 9 initial scales and their subscales was tested for reliability. Items that did not contribute to reliability or those that unbalanced the final StP-II were removed. In three cases (ATA, CNFU and DOSPERT-R) whole subscales were removed as they were unreliable in the present experiment. In the case of ATA only the economic dimension was retained. In case of DOSPERT-R, only financial and ethical domains were kept for further analysis. In CNFU, Avoidance of Similarity emerged as a separate construct in a subsequent series of exploratory factor analyses, while Unique Consumer Behaviour proved to be unreliable and was removed. The subscales of Creative Choice and Unpopular Choice were rolled into a single subscale labelled Choice (the latent structure was later established through exploratory factor analysis). Each subscale and their subsequent subscales were tested using the Main and the Holdout samples. The results of the reliability tests are presented in Table 1. The reliability scores range from adequate (.767) to high (.909), with the reliability of the whole scale still high (.942 on Holdout sample and .948 on Main sample).

**Table 1:** Reliability scores for susceptibility to persuasion scale—II on holdout (n = 278) and main (n = 500) samples.

Item	[Items]	a_s_ (Holdout)^a^	n_h_	a_s_ (Main)^b^	n_m_
Premeditation	6	.886	262	.884	476
Consistency	6	.887	259	.889	476
Sensation Seeking	6	.800	265	.782	471
Self-Control	6	.856	263	.855	467
Social Influence	6	.864	264	.861	462
Similarity	4	.899	265	.883	479
Risk Preferences	6	.900	258	.909	472
Attitudes towards Advertising	4	.780	265	.822	481
Need for Cognition	6	.865	263	.893	460
Uniqueness	4	.767	267	.795	479
Full StP-II reliability	54	.942	198	.948	350

Note.

^a^ Standardized Cronbach Alpha for the Holdout sample.

^b^ Standardized Cronbach Alpha for the Main sample.

In addition to the development of full StP-II scale, a brief version of the scale (StP-II-B) was also constructed and tested for reliability. The test results are presented in Table 2. StP-II-B sacrifices preciseness (i.e. in subscales containing further subscales, the latter are rolled into a single construct / subscale) and a small amount of reliability (which ranges from .747 to .912) for the sake of brevity and ease of application.

**Table 2:** Reliability scores for susceptibility to persuasion scale—II (Brief) on holdout (n = 278) and main (n = 500) samples.

Item	[Items]	a_s_ (Holdout)^a^	n_h_	a_s_ (Main)^b^	n_m_
Premeditation	3	.849	267	.842	487
Consistency	3	.813	269	.831	486
Sensation Seeking	3	.777	268	.747	479
Self-Control	3	.800	270	.768	483
Social Influence	3	.901	270	.895	476
Similarity	3	.880	272	.876	484
Risk Preferences	3	.912	261	.911	482
Attitudes towards Advertising	3	.804	269	.830	488
Need for Cognition	3	.833	273	.832	482
Uniqueness	3	.748	268	.826	486
Full StP-II reliability	30	.910	226	.917	389

Note.

^a^ Standardized Cronbach Alpha for the Holdout sample.

^b^ Standardized Cronbach Alpha for the Main sample.

Each initial subscale used to construct StP-II was individually explored using factor analysis (on the Holdout sample) and each of their subscales tested for reliability. Their initial structures were confirmed with adequate loadings and good reliability. The results of the analysis are presented in S1 Table in the Supplemental Materials Section.

### Factor analysis of StP-II on the main sample

The experimental data were screened for univariate outliers. The minimum amount of data for factor analysis was satisfied, with final sample sizes of 500 (Main) and 279 (Holdout) and universally high factor loadings of the Susceptibility to Persuasion–II subscales [68].

The factor structure of the 54 remaining items in StP-II scale items was examined. Several factorability criteria were used. On the Main sample, the Kaiser-Meyer-Olkin measure of sampling adequacy was .934, above the recommended value of .5. Bartlett’s test of sphericity was significant (χ^2^_1431_ = 18040.91, *p* < .001). All communalities were above .321, with 43 items above .5 and 25 above .6.

Out of the two most commonly used factoring techniques Principal Axis Factoring was employed. The Maximum Likelihood method assumes multivariate normal distribution of the variable (e.g. [69]) and that condition was not fulfilled in our data, most notably in DOSPERT-R where respondents self-reported to be highly risk averse, thus skewing the distribution. Direct Oblimin rotation was used, as we assumed that certain factors would share variance. Initial eigenvalues showed that the first factor explained 27% of the variance, the second 9%, the third factor 8% of the variance, the fourth, fifth and sixth factor 3% of the variance, the seventh, eighth and ninth factor approximately 2% of the variance and the tenth factor 1% of the variance. The ten-factor solution (of subscales with eigenvalues > 1) explained 59% of the variance. Factor loadings ranged from .452 to .819 with minimal cross-loading. All factor loadings for full StP-II scale are listed in S1 Table in the Supplemental Materials Section.

The group of items we named Premeditation explained most of the variance (27%). Individuals who are able to foresee the future consequences of their actions weigh their options carefully before committing to a certain course of action. Thus, they may be more resistant to the influence of persuasive techniques. The factor named Consistency explained 9% of the variance; individuals with high scores in this factor feel very strong need for consistency and structure, so they may be more susceptible to persuasion once they initially committed. Finally, the third factor, we named Sensation Seeking, explained 8% of the variance. Individuals seeking novel and intense experiences will be more likely to commit to an action if they perceive it as viscerally enticing.

### Reliability across samples

To check for repeatability of StP-II, we ran exploratory factor analysis on the Holdout group (n = 279). Principal Axis Factoring with Oblimin rotation was again used. The Kaiser-Meyer-Olkin measure of sampling adequacy was .862. Bartlett’s test of sphericity was significant (χ^2^_1431_ = 7155.53, *p* < .001). A ten-factor structure emerged with the top three factors in the same order as in the Main group. Initial eigenvalues showed that the first factor explained 26% of the variance, the second 10%, and the third factor 7% of the variance. The solution explained 60% of the variance. The 54 items loaded in the same factors, although the order of the lesser factors was slightly changed. Independent sample t-tests were run across the subscale means to establish whether there were differences between the two groups. No statistically significant differences were found between the answer means in the two samples (cf. Table 3), leading us to infer that the scale factor structure is robust.

**Table 3:** Means, standard deviations, extracted variance, T-Test values, and significance of differences in StP-II main (n = 500) and holdout (n = 279) groups.

	Subscale	Mean	SD	Extracted Variance [%]	t	p
Main						
	Premeditation	4.15	1.33	26.7		
	Consistency	5.05	1.21	9.4		
	Sensation Seeking	4.78	1.24	6.1		
	Self-control	3.97	1.42	3.5		
	Social Influence	4.45	1.38	3.2		
	Similarity	3.73	1.61	3.0		
	Risk Preferences	2.79	1.65	2.4		
	Att. to Advertising	4.52	1.40	1.9		
	Cognition	4.15	1.45	1.5		
	Unique Choice	4.34	1.38	1.2		
Total				58.9		
Holdout						
	Premeditation	4.28	1.35	25.7	-1.317	.188
	Consistency	5.13	1.17	9.6	-0.952	.341
	Sensation Seeking	4.74	1.33	6.5	0.472	.637
	Self-control	3.97	1.46	3.0	0.021	.983
	Social Influence	4.55	1.42	1.6	-0.947	.344
	Similarity	3.71	1.65	3.7	0.221	.826
	Risk Preferences	2.88	1.69	4.5	-0.716	.474
	Att. to Advertising	4.54	1.31	2.0	-0.127	.899
	Cognition	4.20	1.42	2.2	-0.464	.643
	Unique Choice	4.38	1.33	1.2	-0.366	.714
Total				60.1		

### Confirmatory factor analysis of StP-II

To test the latent structure of the full StP-II scale (54 items, 10 subscales) that was established through exploratory factor analysis in Section “Factor Analysis of StP-II on the Main Sample”, we ran confirmatory factor analysis on the holdout sample (n = 279). The determination of the model fit will be based on multiple fit indices. Although chi-square statistic was used to determine fit, it is a generally volatile test influenced by diverse set of factors (e.g. with larger sample sizes, the test is almost always significant, suggesting lack of fit; [70]), so it cannot be used as a sole indicator of fit [69]. Brown [69] suggests reporting an index of absolute fit, an index for fit adjusting for model parsimony, and an index for comparative or incremental fit. Thus, we report the standardized root mean square residual (SRMR), the root mean-square error of approximation (RMSEA) and the comparative fit index (CFI).

The holdout sample was screened for outliers and the cases containing missing data (n = 9) were studied. Outliers were removed at the previous stage of analysis, and Little’s missing completely at random (MCAR) test yields a non-significant chi square result (χ^2^_16105_ = 15674.11, n.s., *p* = .992) indicating that the missing values have appeared randomly in the holdout sample. We thus approximated the missing values using the full information maximum likelihood (FIML) method as described by Schafer and Graham [71], and Allison [72]. Note that the latter refers to FIML as the ‘direct ML’.

The model converged normally after 148 iterations and yielded a significant chi-square test result (χ^2^_1299_ = 2216.25, p < .001). However, the χ^2^ / df ratio at 1.71 was lower than 2 indicating that the model was a good fit. Standardized Root Mean Square Residual indicated good fit (SRMR = .066) at less than .08 [73]. The Root Mean Square Error of Approximation (RMSEA = .050) was sufficiently low (< .06; [73]) with the confidence interval of .047 to .054, to indicate good fit. The Comparative Fit Index (CFI = .894) was lower than expected value range of .90 –.95, although this is acceptable, considering the complexity of the model and a relatively small sample [74].

All the 54 Items loaded into the appropriate subscales (standardised loadings min: .519, max: .901). The full factor loading S4 Table is presented in the Supplemental Materials Section.

### Confirmatory factor analysis of StP-IIB

The holdout sample (n = 279) was again used to test the latent structure of StP-IIB (30 items, 10 subscales). Missing values were again estimated using direct ML method; and the model again converged normally after 109 iterations. The chi-square result was again significant (χ^2^_360_ = 525.28, p < .001) with an even lower ratio of 1.46. All the fit indices: SRMR = .045, RMSEA = .041 (95% CI [.033 - .048]), and CFI = .963 indicated a good fit. Note that the Comparative Fit Index now rose above the acceptable level, indicating that the complexity of the model combined with the low sample size clearly impacts it.

All the items loaded in the appropriate subscales with the lowest overall factor loading of .637 and highest loading of .902. The full factor loading S5 Table is presented in the Supplemental Materials Section.

## Method 2

### Participants

This study was advertised on the BBC Future website in October 2014. Our participants self-selected to fill out an online questionnaire. The data that were collected for this study were part of a larger victimization study. Participants did not receive monetary compensation but any that opted in received a personalized email discussing their results (n = 2131). Raw sample used for analysis contained 10493 responses. Out of those, 3884 responses were discarded as they failed to answer more than 50% of the StP-II items. The final sample size for Study 2 was thus 6609 responses. To control for order effects, the StP-II items were randomised in each iteration of the survey.

In the measured group, age was negatively skewed, with most respondents reporting to be 41–50 years old (23%) with another 22% aged between 51 and 60. Males were over-represented at 71% (n = 4588; females: 29%; n = 1840). In education, most respondents (76%) self-reported to have at least a University degree (36%), a Masters or a professional degree (30%) or a Doctoral degree (10%). Most respondents (57%; n = 3641) reported that they were in a long-term relationship or married. Most respondents (38%, n = 2492) indicated that they were employed in a private sector.

### Design

A series of correlations and regression analyses were run on the data to establish the construct validity of StP-II. In addition, StP-II was analysed using exploratory factor analysis and re-tested for reliability (using Cronbach’s Alpha). Full scale and its items is listed in the Supplemental Materials Section under S1 Appendix.

## Dependent variables

The dependent variables were derived from 10 questions describing 10 fraudulent schemes. The schemes were assembled from American National Consumer League’s Fraud Center whitepaper on fraud Trends [75], the UK Office of Fair Trading report on psychology of fraud [3] and respondent-reported scams from previous studies (e.g. [5, 10, 12]). Respondents were asked to mark how plausible they found each of the 10 schemes on a Likert type scale of 1 to 7, where 1 was completely implausible and 7 was completely plausible. Overall plausibility (DV) is an amalgamated mean of particulate plausibility scores for each scheme. Descriptive statistics for each scheme are reported in Table 4.

**Table 4:** Descriptives for overall and plausibility of individual schemes (n = 6609).

	n (missing)^a^	Mean	SD
[ACCOMODATION] An accommodation ad, with very reasonable conditions (for example, rent about half the usual amount).	1660 (4949)	2.56	1.77
[AUCTION] An auction with a low price for a very desirable item.	2562 (4047)	2.43	1.72
[BOILER ROOM] A call from a broker offering you an insider tip on some good value stock.	1733 (4876)	1.76	1.40
[COMPUTER HIJACK] An email or webpage advertising free security sweep or free antivirus scan.	5335 (1274)	2.01	1.63
[COUNTERFEIT GOODS] An online store selling genuine goods for a fraction of the usual price.	3592 (3017)	2.27	1.64
[IDENTITY THEFT] An email from the system administrator or the bank manager saying you need to provide your login details or bank access codes.	4700 (1909)	1.53	1.28
[LOTTERY SCAMS] An email notifying you that you won an online lottery.	4262 (2347)	1.18	0.77
[ADVANCE FEE FRAUD] An email telling you are about to receive a large windfall (inheritance, dormant bank account, free loan, EU development funds).	4157 (2452)	1.16	0.72
[LONELY HEART SWINDLES] An unknown person contacting you, looking for companionship or a bit of fun.	4080 (2529)	1.36	1.02
[PYRAMID SCHEME] An invitation to participate in a marketing event, where you could quickly become rich without any investment on your part.	3221 (3388)	1.43	1.03
[OVERALL] General plausibility	6268 (341)	1.74	1.06

Note. Question: Have you received any of the following communications in the past three years? How believable did they seem to you when you first saw them? Please answer on a scale 1 to 7, where 1 is not at all believable and 7 is perfectly fine.

For example, if you wanted to rent a property and have come across an ad that offered very reasonable conditions, almost too reasonable, did you think: "This is too good to be true. It is a scam for sure"? Then mark 1. If you weren't sure one way or the other, mark 4. If the ad seemed perfectly legitimate and you didn't suspect anything initially, then mark as 7.

^a^ Values in (brackets) denote missing or non-applicable values. In addition to marking plausibility, respondents were asked to mark whether they have ever come across this type of scheme.

Table 4 shows that individual and overall plausibility scores are positively skewed. Most respondents find prevalent fraudulent communication suspect. Out of 6609 respondents approximately 37% (n = 2288) found all scenarios presented highly implausible.

Responded was the second dichotomous dependent variable that indicated whether the respondents interacted with scammers across the ten measured schemes. It was coded as 1 = not responded (n = 3858, 58%), and 2 = responded to at least one scheme in the past three years (n = 2751, 42%).

Lost was the third dichotomous dependent variable. It shows whether the respondents lost utility to any of the presented ten schemes (listed in Table 4). It was coded as 1 = did not lose any utility (n = 5152, 78%), and 2 = lost utility to at least one of the fraudulent offers (n = 1457, 22%).

Note that the groups are not of equal sizes, but that is acceptable in the general- and generalized linear models, for a sample of this size [68]. Likewise, an analysis of variance is robust enough against bivariate variables [76].

## Independent variables

There were 11 independent variables, representing the 10 subscales of StP-II, with an additional variable “StP-II” representing the mean score of the whole scale (the aggregate variable StP-II was not entered in any statistical model together with the subscales of which it is comprised). The descriptives and reliability of the independent variables are listed in Table 5.

**Table 5:** Descriptives and reliability of susceptibility to persuasion—II scale in study 2 (n = 6609).

	Mean	S.E.	SD	α	α_s_	Items
Premeditation	2.70	0.013	1.09	.793	.793	6
Consistency	4.01	0.015	1.19	.813	.813	6
Sensation Seeking	4.32	0.015	1.24	.682	.679	6
Self-control	3.93	0.014	1.17	.734	.733	6
Social Influence	3.30	0.012	1.01	.719	.715	6
(Need for) Similarity	4.56	0.017	1.35	.818	.817	4
Risk Preferences	1.58	0.009	0.72	.645	.671	6
Att. to Advertising	2.79	0.016	1.29	.801	.806	4
Cognition	2.60	0.012	0.99	.680	.685	6
Unique Choice	3.81	0.016	1.27	.673	.676	4
StP-II (Overall)	3.32	0.006	0.50	.810	.820	54

Note. Letters α and αs denote Cronbach α and Cronbach α standardised values respectively.

Table 5 indicates reasonably normal distribution of responses in most subscales with risk preferences positively skewed, indicating that individuals are risk averse in general when it comes to finances and ethics. The reliability of particulate scales ranges from moderate to high (.671 to .817) with the reliability of the whole scale at .820.

### Procedure

The questionnaire was delivered online and consisted of 6 sequential parts:

- Introduction
- Demographics (Age, Gender, Occupational Status, IT Knowledge, Relationship status, Education).
- Scam compliance section (A series of questions connected to scam compliance and its stages)
- Susceptibility to Persuasion–II scale (54 Items)
- Follow-up questions (demand characteristics control, request to contact again, email).
- Debriefing.

## Results 2

### Exploratory factor analysis of StP-II

An exploratory factor analysis was run to establish the validity of the factor structure across samples. Principal Axis Factoring was used with Oblimin Rotation. The factorability criteria of StP-II were satisfied in Study 2, with the Kaiser-Meyer-Olkin measure of sampling adequacy of .875. Bartlett’s test of sphericity was significant with χ^2^_1431_ = 77755.15, *p* < .001. The solution explained 42% of the variance and kept the same order of factors. The analysis of the 54 items in StP-II yielded a comparable structure, with the same subscales and sub-subscales as in Study 1. The factor loadings ranged from .149 to .848 with most over .550 and very few cross-loading items. Detailed factor structure is reported in S2 Table in the Supplemental Materials Section.

### Construct validity

In order to measure the construct validity across samples, we ran confirmatory factor analysis on the whole sample in the second study. We first analysed the missing data. Little’s MCAR test yields insignificant results (χ^2^_67612_ = 58991.6, n.s, p = 1.000), therefore the missing data is distributed randomly and maximum likelihood estimation method was used to replace the missing values. We used the same fit indices as in Section “Confirmatory Factor Analysis of StP-II”.

The model converged normally in 113 iterations. The chi-square result was again significant (1299 = 7180.25, p < .001) with a ratio of 5.53. All the fit indices, SRMR = .032, RMSEA = .026 (95% CI [.026 - .027]), and CFI = .938, indicated an excellent fit. All latent variables loaded into the correct subscales with factor loadings ranging from 0.328 to 0.844.

### Applicability of StP-II to scam compliance

We first screened the Study 2 response data for outliers and subsequently removed 660 responses, ending up with a sample size of 5949 in the plausibility context. The removal of outliers was done in five steps, based on excluding variable data that were 2.5 standard deviations above or below the mean values. There were no outliers 2.5 standard deviations from the mean in responded condition, and there were 17 outliers (at 2.5 SD) in the lost condition. They were removed in five steps.

To establish construct validity, a series of Spearman rho bivariate correlations were first run across overall plausibility, and overall StP-II, with its subscale means. All correlations were statistically significant, but low, ranging from r_s_ = -.045 (general plausibility * need for similarity, n = 5627, p = .001) to r_s_ = .246 (general plausibility * overall StP-II, n = 5631, p < .001). Note that only the need for similarity was negatively correlated to general plausibility. A full list of correlations is listed in S3 Table in the Supplemental Materials Section.

Before running OLS regression we also evaluated the bivariate Pearson correlations between the subscales of the independent variables and performed model diagnostics. There were five significant correlations above .300 in the present sample. Out of 55 correlations, 50 were statistically significant at p < .001, 2 at p < .05 and three were non-significant. None of the variables were removed from further analysis, but additional collinearity diagnostics were run on them. There was a high condition index in the last, 11^th^ dimension of the model (29.96), but it did not rise above the cut-off value of 30 (cf. [68]). In addition, only one variable in this dimension carried a variance proportion greater than .5 (Similarity = .51). Thus, there was no collinearity issue in the present sample allowing us to proceed with the analysis.

Thereafter a series of regression analyses was employed to further evaluate the construct validity of StP-II. Due to the highly skewed distribution of overall plausibility scores (original DV), the model residuals in ordinary least squares (OLS) regressions predicting it were heteroscedastic. This violates the assumptions of OLS linear models. To deal with this issue, we created a dichotomous variable that denoted whether respondents found any of the scenarios plausible (participants who rated the plausibility of any of the 10 scenarios presented to them higher than 4 were coded as 1 = “cautious participants” [n = 4937] and 2 = “credulous participants” [n = 1112] Consequently we fit a series of logistic regression models using Plausible, Responded and Lost as the dichotomous dependent variables.

All three models were significant, with Plausible χ^2^_10_ = 350.62, *p* < .001; Nagelkerke’s R^2^ = .10, Responded χ^2^_11_ = 335.44, *p* < .001; Nagelkerke’s R^2^ = .07, and Lost χ^2^_12_ = 1461.61, *p* < .001; Nagelkerke’s R^2^ = .31. The odds of being a “credulous participant” increased with increasing scores on 9 out of 10 StP-II subscales–while controlling for the effects of the other subscales. Correspondingly, the odds of interacting with a scammer were influenced by 5 out of 9 StP-II subscales and by finding an offer plausible. Finally, the likelihood of losing utility to a fraudulent scheme was significantly influenced by 3 out of 10 StP-II subscales, and strongly influenced by finding a fraudulent offer plausible, and previously interacting with scammers (cf. Table 6).

**Table 6:** Logistic regression models for StP-II salience across the stages of universal scam compliance.

	*Model 1*: *Plausible* *(n = 5864)*			*Model 2*: *Responded* *(n = 6514)*			*Model 3*: *Lost* *(n = 6514)*		

Variable	B	S.E.	Wald	B	S.E.	Wald	B	S.E.	Wald
Premeditation	-.02	0.04	0.41	.00	0.03	0	0	0.04	0
	[0.98]			[1.00]			[1.00]		
Consistency	.06	0.03	3.93**	.06	0.02	6.84**	.02	0.03	0.56
	[1.07]			[1.06]			[1.02]		
Sensation Seeking	.13	0.03	18.77***	.02	0.02	0.84	-.05	0.03	3.41*
	[1.14]			[1.02]			[0.95]		
Self - Control	.21	0.04	34.24***	.02	0.03	0.79	0	0.03	0.02
	[1.23]			[1.02]			[1.00]		
Social Influence	.31	0.04	62.53***	.09	0.03	10.63**	.04	0.04	1.4
	[1.37]			[1.10]			[1.04]		
Similarity	.07	0.03	4.70**	.03	0.02	1.8	-.02	0.03	0.29
	[1.07]			[1.03]			[0.98]		
Risk preferences	.09	0.05	3.51*	.09	0.04	5.11**	-.02	0.05	0.15
	[1.10]			[1.09]			[0.98]		
Att. To Advertising	.07	0.03	5.64**	0	0.02	0.02	-.01	0.03	0.17
	[1.07]			[1.00]			[0.99]		
Cognition	.14	0.04	11.62**	.07	0.03	5.68**	.08	0.04	3.72*
	[1.15]			[1.08]			[1.08]		
Uniqueness	.18	0.03	28.02***	.15	0.02	40.6***	.07	0.03	4.66**
	[1.19]			[1.17]			[1.07]		
Plausible				.8	0.06	161.64***	.34	0.08	19.98***
				[2.22]			[1.41]		
Responded							2.47	0.08	956.68***
							[11.76]		
Constant	-5.91	0.33	323.72***	-3.10	0.23	180.42***	-5.98	0.32	353.34***
	[0]			[.045]			[0]		
Model χ^2^	350.62***			335.44***			1461.61***		
	10			11			12		
Nagelkerke R^2^	.10			.07			.31		

Note. Entries (b) are unstardardized logistic regression coefficients, (S.E.) are standard errors, odds ratios are in square brackets.

* p < .1

** p < .05

*** p < .001

Two of the three models have a relatively low predictive strength, indicating that while susceptibility to persuasion plays a significant role in plausibility and responding, other currently unassessed factors play a role as well.

Because dichotomous variables contain less statistical information than continuous ones, heteroscedasticity-consistent standard error estimators in OLS regression (i.e., robust regression) were employed to confirm the results of the logistic regression analyses (cf. [77, 78]). Participants with higher StP-II scores had higher overall plausibility scores (B = 0.436, *t*(5628) = 15.8, 95% CI [0.381–0.489], *p* < .001, R^2^ = .05).

We also entered all StP-II subscales into a robust multiple regression model, predicting overall plausibility. This model was statistically significant (F_10, 5544_ = 32.9, *p* < .001, R^2^ = .06). The predictive power of the individual subscales–while controlling for the effects of the other subscales–closely matched those in the logistic regression model, except for the “need for consistency”, which was not a significant predictor in this model. However, given that the model residuals were *highly* heteroscedastic, even robust regression models need to be interpreted with caution. Due to this, the logistic regression models presented above are probably more reliable.

In addition, we conducted a multivariate analysis of variance with Plausible, Responded and Lost as the dichotomous DVs and StP-II mean score as an IV. The main effects were significant with Plausible F_1,6607_ = 122.30, p < .001, α = 1.0, η^2^ = .02, Responded F_1,6607_ = 120.98, p < .001, α = 1.0, η^2^ = .02, and Lost F_1,6607_ = 52.93, p < .001, α = 1.0, η^2^ = .01. Note that using dichotomous DVs is a violation of general linear model assumptions. However, several researchers have shown that the general linear model is robust enough to show appropriate results if the sample proportions for cells are between .25 and .75, and the degrees of freedom for error are larger than 40 [79–81]. In the present analysis, the proportions are: Plausible = .27. Responded = .71, Lost = .28, with 6607 degrees of freedom for error. Also note that some researchers show that regardless of proportions, big enough sample sizes the differences in ANOVA results between continuous and dichotomous variables are negligible [82].

We noticed that (lack of) premeditation was not a significant regressor in general scam compliance. Therefore, we ran a series of multivariate ANOVA’s with three sets of ten dependent variables: Plausible for each of the ten measured fraud categories, Responded for the same categories, and Lost utility (indicating who lost utility in a specific type of fraud). In all three models, the independent variables were the StP-II subscales. In the Responded model, (lack of) premeditation was a significant predictor of respondents interacting with those who commit identity theft (at p < .1), advance fee fraud (at p < .05), and romance scams (at p < .05). In the Lost utility model, (lack of) premeditation was a significant regressor in romance scams (at p < .05).

## Discussion

We have presented the development of StP-II, an amalgamated scale measuring susceptibility to persuasion. This refines and extends the only existing scale of this scope, namely the first version of the StP scale developed by Modic and Lea [5]. The underlying constructs described by the subscales in StP-II have been extensively tested and validated over many years. In addition, a reliable and valid latent structure in both the full and brief versions of StP-II emerged.

The exploratory factor analysis and reliability testing of the respondent data yielded two scales. StP-II in full contains 10 subscales, spanning over 54 items. Three subscales contain further subscales offering a more precise insight into specific constructs. StP-II-B is a brief version of the scale with 10 subscales (30 items), which still measures first order factors, but removes second order constructs for the sake of brevity and the ability to conduct quick exploratory diagnostics. Both scales have proven to be reliable and repeatable. Reliability testing across Main and Holdout samples showed no significant differences between groups.

Construct validity was successfully established through three sets of confirmatory factor analyses (on the holdout sample in study 1 and on the whole sample in study 2). Each time, the fit of the latent structure was confirmed with moderate to good indicators and factor loadings. The overall scale significantly interacted with all three stages of scam compliance, with low effect sizes, but high observed power.

In previous work, we showed that Internet fraud is a staged process, consisting of three stages, that is plausibility, interaction with a fraudster, and losing utility to fraud [5, 10, 16]. We reconfirmed these findings by entering the previous stage of scam compliance in a logistic regression model measuring the next stage. In all cases, the regressor was significant. Responding is significantly positively influenced by finding a fraudulent offer plausible, and losing utility is strongly predicted by finding a scam plausible and responding to it. Note that in the lost model, the effect size increases substantially when responding is introduced. In addition, note that previous research into scam compliance, for example by Shadel [15] theoretically postulates that becoming a victim of fraud is process consisting of four stages (taking re-victimisation into account, as the final stage). Our model empirically confirms these findings, while neglecting to measure re-victimisation.

Further validation of the StP-II impact on scam compliance showed that nine out of ten StP-II subscales were significant regressors in predicting overall plausibility, while five subscales were salient in predicting an interaction with scammers and three significantly predicted losing utility to fraud. Premeditation was the only construct that did not significantly influence any stage of overall scam compliance. However, (lack of) premeditation is a significant predictor of responding and using utility in several particulate categories of Internet fraud. As far as plausibility is concerned, one reasonable explanation for non-significant interaction would be that since consideration of future consequences (i.e. premeditation) has been shown to be influenced by clarity and frequency of an event [83], and since Internet Fraud in its many forms is a low-probability event [4, 6, 12, 16], it is not surprising that individuals would have a hard time predicting whether a given fraudulent scenario is plausible or not. Further in-depth research into construct validation of StP-II would still be warranted and it is being conducted at the time of this writing.

There is a low significant negative correlation between the need for similarity and overall plausibility, indicating that plausibility decreases when respondents want to be similar to their peers. This is also in line with the general attitude towards fraud. Individuals generally tend to err on side of caution when it comes to activity that may harm them (e.g. [84, 85]) and thus pick a safer option in a gamble, when they want to make choices that follow the consensus. The skewed scores in the StP-II subscale measuring risk preferences also empirically indicate that individuals are likely to be risk averse in conditions of gain, regardless of the context as first shown by Kahneman and Tversky [86].

The correlations across general scam compliance and StP-II subscales are significant, but low. In addition, all regression models but one (losing utility) had only modest predictive power. Both findings indicate that there is a significant relationship between the constructs, but at the same time, they are disparate. Many other factors influence scam compliance, such as age, gender, personality traits [6, 9], context [28, 29], among others. The goal of the present paper is not to establish that scam compliance can be completely explained by StP-II, but that StP-II plays a modest but vital part in our understanding of behaviour modification techniques, regardless of context. In addition, scam compliance constructs and StP-II are sufficiently strongly related that the interaction between the two can be used to cross-validate a scale of susceptibility to persuasion.

### Limitations

There is a potential issue of respondents answering surveys in ways that they believe will gain approval of the experimenter (i.e. social desirability bias). Joinson [87] shows that respondents who fill out anonymised surveys online report lower levels of social desirability and anxiety compared to pen and paper administration of same tests. Booth-Kewley, Edwards [88] showed that the perception of being anonymous influenced the levels of social desirability in respondents when they were comparing responses across pen and paper, and computer based testing. We argue that our online experimental design was sufficiently anonymised to lower the impact of social desirability. A similar issue to social desirability bias is the need of respondents to present themselves in the best possible light and answer accordingly (i.e. self-presentation bias). Buchanan and Smith [89] showed that the self-presentation bias was also significantly reduced by the levels of anonymity.

### Further research

This foundation paper aims to establish StP-II as a viable scale to measure persuadability. In this paper we did not demonstrate all the contexts StP-II is applicable to; instead, we focused on scam compliance across different Internet fraud schemes. Thus, based on the current data, we cannot conclusively state that StP-II is a viable instrument across multiple contexts. Nonetheless, our results clearly show that the scale shows great promise in its function. Moreover, additional research is currently being conducted by us and others to further develop the scale across diverse contexts from personnel screening to establishing the likelihood of falling for an Internet scam. Thus, the susceptibility to persuasion II scale is a modular, reliable and valid scale that has a potentially wide audience. We hope we have made you like our research.

## Supporting information

S1 Appendix Susceptibility to persuasion—II scale items. (DOCX)

S1 Table Factor loadings and communalities based on a Principal Axis Factoring with Oblimin Rotation for 54 items from susceptibility to persuasion—II scale on main sample (n = 500). (DOCX)

S2 Table Factor loadings for susceptibility to persuasion scale using Principal Axis Factoring with Oblimin Rotation (n = 6609). (DOCX)

S3 Table Spearman rho correlations between plausibility and StP-II with its subscales. (DOCX)

S4 Table Standardised factor loadings / Correlations across items in factors for StP-II (n = 279) in confirmatory factor analysis. (DOCX)

S5 Table Standardised factor loadings / Correlations across items in StP-IIB factors (n = 279) in confirmatory factor analysis. (DOCX)
