# Supplementary material for: We will make you like our research: The development of a susceptibility-to-persuasion scale
Source: PLoS One. 2018 Mar 15;13(3):e0194119. doi: 10.1371/journal.pone.0194119 (PMC5854354; doi:10.1371/journal.pone.0194119)
Supplement: S1 Appendix — (DOCX) [file pone.0194119.s001.docx]

Table S1 Appendix. Susceptibility to Persuasion - II Scale Items

| [B]^a^ | Item |
| --- | --- |
| Premeditation | |
| [B] | I only act to satisfy immediate concerns, figuring the future will take care of itself. |
|  | My behavior is only influenced by the immediate (i.e., a matter of days or weeks) outcomes of my actions. |
|  | I generally ignore warnings about potential future problems because I think the problems will be resolved before they reach crisis level. |
| [B] | I think that sacrificing now is usually unnecessary since future outcomes can be dealt with at a later time. |
| [B] | I only act to satisfy immediate concerns, figuring that I will take care of future problems that may occur at a later date. |
|  | Since my day to day work has specific outcomes, it is more important to me than behaviour that has distant outcomes. |
| Consistency | |
|  | It is important to me that those who know me can predict what I will do. |
|  | I want to be described by others as a stable, predictable person. |
| [B] | The appearance of consistency is an important part of the image I present to the world. |
| [B] | An important requirement for any friend of mine is personal consistency. |
|  | I want my close friends to be predictable. |
| [B] | I make an effort to appear consistent to others. |
| Sensation Seeking | |
| *Novelty* | |
| [B] | I would like to travel to places that are strange and far away. |
| [B] | I would have enjoyed being one of the first explorers of an unknown land. |
| [B] | If it were possible to visit another planet or the moon for free, I would be among the first to sign up. |
| *Intensity* | |
|  | If I were to go to an amusement park, I would prefer to ride the rollercoaster or other fast rides. |
|  | In general, I work better when I'm under pressure. |
|  | I like the feeling of standing next to the edge on a high place and looking down. |
| Self-control | |
|  | I have a hard time breaking bad habits. |
| [B] | I say inappropriate things. |
| [B] | I do certain things that are bad for me, if they are fun. |
|  | Pleasure and fun sometimes keep me from getting work done. |
|  | I have trouble concentrating. |
| [B] | Sometimes I can’t stop myself from doing something, even if I know it is wrong. |

Note. Continued on the next page. Items marked with * are reverse scored. a [B] denotes the brief version of the scale (StP-II-B).

Table S1 Appendix (Continued)

| Social Influence | |
| --- | --- |
| *Normative* | |
| [B] | When buying products, I generally purchase those brands that I think others will approve of. |
| [B] | If other people can see me using a product, I often purchase the brand they expect me to buy. |
| [B] | I achieve a sense of belonging by purchasing the same products and brands that others purchase. |
| *Informative* | |
|  | If I have little experience with a product, I often ask my friends about the product. |
|  | I often consult other people to help choose the best alternative available from a product class. |
|  | I frequently gather information from friends or family about a product before I buy. |
| Similarity | |
| [B] | When a product I own becomes popular among the general population, I begin to use it less.* |
| [B] | I often try to avoid products or brands that I know are bought by the general population.* |
|  | As a rule, I dislike products or brands that are customarily bought by everyone.* |
| [B] | The more commonplace a product or brand is among the general population, the less interested I am in buying it.* |
| Risk Preferences | |
| *Financial* | |
| [B] | Betting a day’s income at the horse races. |
| [B] | Betting a day’s income at a high-stake poker game. |
| [B] | Betting a day’s income on the outcome of a sporting event. |
| *Ethical* | |
|  | Passing off somebody else’s work as your own. |
|  | Revealing a friend’s secret to someone else. |
|  | Leaving your young children alone at home while running an errand. |
| Attitudes towards advertising | |
| [B] | Advertising is essential. |
|  | In general, advertising results in lower prices. |
| [B] | Advertising helps raise our standard of living. |
| [B] | Advertising results in better products for the public. |
| Need for Cognition | |
| [B] | I would rather do something that requires little thought than something that is sure to challenge my thinking abilities. |
| [B] | I try to anticipate and avoid situations where there is a likely chance I will have to think in depth about something. |
|  | I like tasks that require little thought once I've learned them. |
| [B] | Learning new ways to think doesn't excite me very much. |
|  | I feel relief rather than satisfaction after completing a task that required a lot of mental effort. |
|  | It's enough for me that something gets the job done; I don't care how or why it works. |
| Need for Unique Choice | |
| [B] | I often combine possessions in such a way that I create a personal image that cannot be duplicated. |
| [B] | I often try to find a more interesting version of run-of-the-mill products because I enjoy being original. |
| [B] | Having an eye for products that are interesting and unusual assists me in establishing a distinctive image. |
|  | When it comes to the products I buy and the situations in which I use them, I have broken customs and rules. |

Note. Instructions: "Please indicate on a scale of 1 to 7, the strength of your agreement with the following statements:"
