# Supplementary material for: We will make you like our research: The development of a susceptibility-to-persuasion scale
Source: PLoS One. 2018 Mar 15;13(3):e0194119. doi: 10.1371/journal.pone.0194119 (PMC5854354; doi:10.1371/journal.pone.0194119)
Supplement: S1 Table — (DOCX) [file pone.0194119.s002.docx]

Table S1. Factor Loadings and Communalities Based on a Principal Axis Factoring with Oblimin Rotation for 54 items from Susceptibility to Persuasion - II Scale on Main Sample (n = 500)

| B^a^ | Item | PR | CS | SS | SC | SI | SM | RI | AD | CG | UN |
| --- | --- | --- | --- | --- | --- | --- | --- | --- | --- | --- | --- |
| B | I only act to satisfy immediate concerns, figuring the future will take care of itself. | .640 |  |  |  |  |  |  |  |  |  |
|  | My behavior is only influenced by the immediate (i.e., a matter of days or weeks) outcomes of my actions. | .617 |  |  |  |  |  |  |  |  |  |
|  | I generally ignore warnings about possible future problems because I think the problems will be resolved before they reach crisis level. | .600 |  |  |  |  |  |  |  |  |  |
| B | I think that sacrificing now is usually unnecessary since future outcomes can be dealt with at a later time. | .592 |  |  |  |  |  |  |  |  |  |
| B | I only act to satisfy immediate concerns, figuring that I will take care of future problems that may occur at a later date. | .762 |  |  |  |  |  |  |  |  |  |
|  | Since my day to day work has specific outcomes, it is more important to me than behavior that has distant outcomes. | .499 |  |  |  |  |  |  |  |  |  |
|  | It is important to me that those who know me can predict what I will do. |  | -.641 |  |  |  |  |  |  |  |  |
|  | I want to be described by others as a stable, predictable person. |  | -.731 |  |  |  |  |  |  |  |  |
| B | The appearance of consistency is an important part of the image I present to the world. |  | -.814 |  |  |  |  |  |  |  |  |
| B | An important requirement for any friend of mine is personal consistency. |  | -.819 |  |  |  |  |  |  |  |  |
|  | I want my close friends to be predictable. |  | -.665 |  |  |  |  |  |  |  |  |
| B | I make an effort to appear consistent to others. |  | -.791 |  |  |  |  |  |  |  |  |
| B | I would like to travel to places that are strange and far away. |  |  | .700 |  |  |  |  |  |  |  |
| B | I would have enjoyed being one of the first explorers of an unknown land. |  |  | .675 |  |  |  |  |  |  |  |
| B | If it were possible to visit another planet or the moon for free, I would be among the first to sign up. |  |  | .753 |  |  |  |  |  |  |  |
|  | If I were to go to an amusement park, I would prefer to ride the rollercoaster or other fast rides. |  |  | .535 |  |  |  |  |  |  |  |
|  | In general, I work better when I'm under pressure. |  |  | .463 |  |  |  |  |  |  |  |
|  | I like the feeling of standing next to the edge on a high place and looking down. |  |  | .527 |  |  |  |  |  |  |  |

Note. Continued on the next page.

Table S1 (Continued)

| B | Item | PR | CS | SS | SC | SI | SM | RI | AD | CG | UN |
| --- | --- | --- | --- | --- | --- | --- | --- | --- | --- | --- | --- |
|  | I have a hard time breaking bad habits. |  |  |  | .786 |  |  |  |  |  |  |
| B | I say inappropriate things. |  |  |  | .571 |  |  |  |  |  |  |
| B | I do certain things that are bad for me, if they are fun. |  |  |  | .716 |  |  |  |  |  |  |
|  | Pleasure and fun sometimes keep me from getting work done. |  |  |  | .588 |  |  |  |  |  |  |
|  | I have trouble concentrating. |  |  |  | .593 |  |  |  |  |  |  |
| B | Sometimes I can’t stop myself from doing something, even if I know it is wrong. |  |  |  | .665 |  |  |  |  |  |  |
| B | When buying products.I generally purchase those brands that I think others will approve of. |  |  |  |  | -.432 |  |  |  |  |  |
| B | If other people can see me using a product, I often purchase the brand they expect me to buy. |  |  |  |  | -.469 |  |  |  |  |  |
| B | I achieve a sense of belonging by purchasing the same products and brands that others purchase. |  |  |  |  | -.529 |  |  |  |  |  |
|  | If I have little experience with a product.I often ask my friends about the product. |  |  |  |  | -.728 |  |  |  |  |  |
|  | I often consult other people to help choose the best alternative available from a product class. |  |  |  |  | -.788 |  |  |  |  |  |
|  | I frequently gather information from friends or family about a product before I buy. |  |  |  |  | -.754 |  |  |  |  |  |
| B | When a product I own becomes popular among the general population, I begin to use it less.* |  |  |  |  |  | -.689 |  |  |  |  |
| B | I often try to avoid products or brands that I know are bought by the general population.* |  |  |  |  |  | -.790 |  |  |  |  |
|  | As a rule, I dislike products or brands that are customarily bought by everyone.* |  |  |  |  |  | -.683 |  |  |  |  |
| B | The more commonplace a product or brand is among the general population, the less interested I am in buying it.* |  |  |  |  |  | -.709 |  |  |  |  |
| B | Betting a day’s income at the horse races. |  |  |  |  |  |  | -.803 |  |  |  |
| B | Betting a day’s income at a high-stake poker game. |  |  |  |  |  |  | -.755 |  |  |  |
| B | Betting a day’s income on the outcome of a sporting event. |  |  |  |  |  |  | -.779 |  |  |  |
|  | Passing off somebody else’s work as your own. |  |  |  |  |  |  | -.503 |  |  |  |
|  | Revealing a friend’s secret to someone else. |  |  |  |  |  |  | -.452 |  |  |  |
|  | Leaving your young children alone at home while running an errand. |  |  |  |  |  |  | -.534 |  |  |  |

Note. Continued on the next page.

Table S1 (Continued)

| B | Item | PR | CS | SS | SC | SI | SM | RI | AD | CG | UN |
| --- | --- | --- | --- | --- | --- | --- | --- | --- | --- | --- | --- |
| B | Advertising is essential. |  |  |  |  |  |  |  | .581 |  |  |
|  | In general, advertising results in lower prices. |  |  |  |  |  |  |  | .550 |  |  |
| B | Advertising helps raise our standard of living. |  |  |  |  |  |  |  | .737 |  |  |
| B | Advertising results in better products for the public. |  |  |  |  |  |  |  | .744 |  |  |
| B | I would rather do something that requires little thought than something that is sure to challenge my thinking abilities. |  |  |  |  |  |  |  |  | .766 |  |
| B | I try to anticipate and avoid situations where there is a likely chance I will have to think in depth about something. |  |  |  |  |  |  |  |  | .672 |  |
|  | I like tasks that require little thought once I've learned them. |  |  |  |  |  |  |  |  | .747 |  |
| B | Learning new ways to think doesn't excite me very much. |  |  |  |  |  |  |  |  | .599 |  |
|  | I feel relief rather than satisfaction after completing a task that required a lot of mental effort. |  |  |  |  |  |  |  |  | .577 |  |
|  | It's enough for me that something gets the job done; I don't care how or why it works. |  |  |  |  |  |  |  |  | .621 |  |
| B | I often combine possessions in such a way that I create a personal image that cannot be duplicated. |  |  |  |  |  |  |  |  |  | .845 |
| B | I often try to find a more interesting version of run-of-the-mill products because I enjoy being original. |  |  |  |  |  |  |  |  |  | .669 |
| B | Having an eye for products that are interesting and unusual assists me in establishing a distinctive image. |  |  |  |  |  |  |  |  |  | .621 |
|  | When it comes to the products I buy and the situations in which I use them, I have broken customs and rules. |  |  |  |  |  |  |  |  |  | .387 |

Note. Items marked with * are reverse scored. a B denotes the brief version of the scale (StP-II-B). Factor loadings < .35 were suppressed.

Legend. PR - Premeditation; CS - Consistency; SS - Sensation Seeking; SC - Self Control; SI - Social Influence; SM - Similarity; RI - Risk Preferences; AD - Attitudes towards Advertising; CG - Need for Cognition; UN – Uniqueness.
