# Supplementary material for: We will make you like our research: The development of a susceptibility-to-persuasion scale
Source: PLoS One. 2018 Mar 15;13(3):e0194119. doi: 10.1371/journal.pone.0194119 (PMC5854354; doi:10.1371/journal.pone.0194119)
Supplement: S2 Table — (DOCX) [file pone.0194119.s003.docx]

Table S2. Factor loadings for Susceptibility to Persuasion Scale using Principal Axis Factoring with Oblimin Rotation (n = 6609)

|  | PR | CN | SES | SC | SIN | SII | SIM | RIF | RIE | ATA | COG | UNI |
| --- | --- | --- | --- | --- | --- | --- | --- | --- | --- | --- | --- | --- |
| PRE1 I only act … itself. | .821 |  |  |  |  |  |  |  |  |  |  |  |
| PRE2 My behavior is only influenced … | .568 |  |  |  |  |  |  |  |  |  |  |  |
| PRE3 I generally ignore warnings … | .509 |  |  |  |  |  |  |  |  |  |  |  |
| PRE4 I think that sacrificing … | .593 |  |  |  |  |  |  |  |  |  |  |  |
| PRE5 I only act .. date. | .787 |  |  |  |  |  |  |  |  |  |  |  |
| PRE6 Since my day to day work … | .278 |  |  |  |  |  |  |  |  |  |  |  |
| CON1 It is important to me … |  | .582 |  |  |  |  |  |  |  |  |  |  |
| CON2 I want to be described ... |  | .667 |  |  |  |  |  |  |  |  |  |  |
| CON3 The appearance of consistency ... |  | .658 |  |  |  |  |  |  |  |  |  |  |
| CON4 An important requirement ... |  | .614 |  |  |  |  |  |  |  |  |  |  |
| CON5 I want my close friends … |  | .634 |  |  |  |  |  |  |  |  |  |  |
| CON6 I make an effort to … |  | .639 |  |  |  |  |  |  |  |  |  |  |
| SSN1 I would like to … |  |  | .564 |  |  |  |  |  |  |  |  |  |
| SSN2 I would have … |  |  | .709 |  |  |  |  |  |  |  |  |  |
| SSN3 If it were possible … |  |  | .668 |  |  |  |  |  |  |  |  |  |
| SSI1 If I were to go to an ... |  |  | .456 |  |  |  |  |  |  |  |  |  |
| SSI2 In general, I work better … |  |  | ^a^ |  |  |  |  |  |  |  |  |  |
| SSI3 I like the feeling of ... |  |  | .434 |  |  |  |  |  |  |  |  |  |
| SCN1 I have a hard time ... |  |  |  | .601 |  |  |  |  |  |  |  |  |
| SCN2 I say inappropriate … |  |  |  | .383 |  |  |  |  |  |  |  |  |
| SCN3 I do certain things ... |  |  |  | .565 |  |  |  |  |  |  |  |  |
| SCN4 Pleasure and fun … |  |  |  | .507 |  |  |  |  |  |  |  |  |
| SCN5 I have trouble … |  |  |  | .353 |  |  |  |  |  |  | .297 |  |
| SCN6 Sometimes I can’t stop … |  |  |  | .574 |  |  |  |  |  |  |  |  |
| SIN1 When buying products … |  |  |  |  | .676 |  |  |  |  |  |  |  |
| SIN2 If other people can see me … |  |  |  |  | .619 |  |  |  |  |  |  |  |
| SIN3 I achieve a sense of ... |  |  |  |  | .601 |  |  |  |  |  |  |  |
| SII1 If I have little experience … |  |  |  |  |  | .778 |  |  |  |  |  |  |
| SII2 I often consult … |  |  |  |  |  | .704 |  |  |  |  |  |  |
| SII3 I frequently gather … |  |  |  |  |  | .824 |  |  |  |  |  |  |
| SIM1 When a product I own ...* |  |  |  |  |  |  | .531 |  |  |  |  |  |
| SIM2 I often try to avoid ...* |  |  |  |  |  |  | .760 |  |  |  |  |  |
| SIM3 As a rule, I dislike ...* |  |  |  |  |  |  | .783 |  |  |  |  |  |
| SIM4 The more commonplace ...* |  |  |  |  |  |  | .750 |  |  |  |  |  |

Note. Continued on the next page.

Table S2 (continued).

|  | PR | CN | SES | SC | SIN | SII | SIM | RIF | RIE | ATA | COG | UNI |
| --- | --- | --- | --- | --- | --- | --- | --- | --- | --- | --- | --- | --- |
| RIF1 ...horse races. |  |  |  |  |  |  |  | .848 |  |  |  |  |
| RIF2 … high-stake poker game. |  |  |  |  |  |  |  | .761 |  |  |  |  |
| RIF3 … a sporting event. |  |  |  |  |  |  |  | .826 |  |  |  |  |
| RIE1 Passing off somebody … |  |  |  |  |  |  |  |  | .367 |  |  |  |
| RIE2 Revealing a … |  |  |  |  |  |  |  |  | .372 |  |  |  |
| RIE3 Leaving your … |  |  |  |  |  |  |  |  | .236 |  |  |  |
| ATA1 Advertising is essential. |  |  |  |  |  |  |  |  |  | .629 |  |  |
| ATA2 In general, advertising … |  |  |  |  |  |  |  |  |  | .614 |  |  |
| ATA3 Advertising helps … |  |  |  |  |  |  |  |  |  | .793 |  |  |
| ATA4 Advertising results … |  |  |  |  |  |  |  |  |  | .810 |  |  |
| COG1 I would rather do something … |  |  |  |  |  |  |  |  |  |  | .646 |  |
| COG2 I try to anticipate … |  |  |  |  |  |  |  |  |  |  | .514 |  |
| COG3 I like tasks … |  |  |  |  |  |  |  |  |  |  | .501 |  |
| COG4 Learning new ways … |  |  |  |  |  |  |  |  |  |  | .379 |  |
| COG5 I feel relief rather … |  |  |  |  |  |  |  |  |  |  | .457 |  |
| COG6 It's enough for me … |  |  |  |  |  |  |  |  |  |  | .290 |  |
| UNI1 I often combine … |  |  |  |  |  |  |  |  |  |  |  | .623 |
| UNI2 I often try to find … |  |  |  |  |  |  | .207 |  |  |  |  | .504 |
| UNI3 Having an eye … |  |  |  |  |  |  |  |  |  |  |  | .764 |
| UNI4 When it comes … |  |  |  |  |  |  |  |  |  |  |  | .202 |

Note. All factors loadings below .2 have been supressed. ^a^ Factor Loading of .149. Legend. PR - Premeditation; CN - Consistency; SES - Sensation seeking (Intensity and Novelty combined); SC - Self-Control; SIN - Social Influence (Normative); SII - Social Influence (Informative); SIM - Need for Similarity; RIF - Risk (Financial); RIE - Risk (Ethical); ATA - Attitude towards advertising; COG - Need for Cognition; UNI - Need for Uniqueness.
