# Supplementary material for: We will make you like our research: The development of a susceptibility-to-persuasion scale
Source: PLoS One. 2018 Mar 15;13(3):e0194119. doi: 10.1371/journal.pone.0194119 (PMC5854354; doi:10.1371/journal.pone.0194119)
Supplement: S3 Table — (DOCX) [file pone.0194119.s004.docx]

Table S3. Spearman rho Correlations Between Plausibility and StP-II with its Subscales

|  | Overall Plausibility | |
| --- | --- | --- |
|  | r_s_ | n |
| Premeditation | .116*** | 5630 |
| Consistency | .074*** | 5629 |
| Sensation Seeking | .095*** | 5627 |
| Self-control | .168*** | 5631 |
| Social Influence | .192*** | 5629 |
| Similarity | -.045**^a^ | 5627 |
| Risk Preferences | .165*** | 5584 |
| Att. to Advertising | .118*** | 5620 |
| Cognition | .147*** | 5631 |
| Unique Choice | .125*** | 5619 |
| Overall mean of StP-II | .246^***^ | 5631 |

Note. *** p < .001, ** p < .05 a p = .00
