# Supplementary material for: We will make you like our research: The development of a susceptibility-to-persuasion scale
Source: PLoS One. 2018 Mar 15;13(3):e0194119. doi: 10.1371/journal.pone.0194119 (PMC5854354; doi:10.1371/journal.pone.0194119)
Supplement: S4 Table — (DOCX) [file pone.0194119.s005.docx]

Table S4. Standardised Factor Loadings / Correlations Across Items in Factors for StP-II (n = 279) in Confirmatory Factor Analysis

|  | Factor | | | | | | | | | | | | |
| --- | --- | --- | --- | --- | --- | --- | --- | --- | --- | --- | --- | --- | --- |
|  | PRE | COG | CONS | S-C | SS | | SI | | RI | | SIM | UNQ | ATA |
|  |  |  |  |  | NOV | INT | NORM | INFO | FIN | ETH |  |  |  |
| PRE1 I only act … itself. | 0.832 |  |  |  |  |  |  |  |  |  |  |  |  |
| PRE2 My behavior is only influenced … | 0.761 |  |  |  |  |  |  |  |  |  |  |  |  |
| PRE3 I generally ignore warnings … | 0.745 |  |  |  |  |  |  |  |  |  |  |  |  |
| PRE4 I think that sacrificing … | 0.791 |  |  |  |  |  |  |  |  |  |  |  |  |
| PRE5 I only act .. date. | 0.773 |  |  |  |  |  |  |  |  |  |  |  |  |
| PRE6 Since my day to day work … | 0.627 |  |  |  |  |  |  |  |  |  |  |  |  |
| COG1 I would rather do something … |  | 0.819 |  |  |  |  |  |  |  |  |  |  |  |
| COG2 I try to anticipate … |  | 0.815 |  |  |  |  |  |  |  |  |  |  |  |
| COG3 I like tasks … |  | 0.670 |  |  |  |  |  |  |  |  |  |  |  |
| COG4 Learning new ways … |  | 0.746 |  |  |  |  |  |  |  |  |  |  |  |
| COG5 I feel relief rather … |  | 0.663 |  |  |  |  |  |  |  |  |  |  |  |
| COG6 It's enough for me … |  | 0.645 |  |  |  |  |  |  |  |  |  |  |  |
| CON1 It is important to me … |  |  | 0.744 |  |  |  |  |  |  |  |  |  |  |
| CON2 I want to be described ... |  |  | 0.741 |  |  |  |  |  |  |  |  |  |  |
| CON3 The appearance of consistency ... |  |  | 0.747 |  |  |  |  |  |  |  |  |  |  |
| CON4 An important requirement ... |  |  | 0.733 |  |  |  |  |  |  |  |  |  |  |
| CON5 I want my close friends … |  |  | 0.787 |  |  |  |  |  |  |  |  |  |  |
| CON6 I make an effort to … |  |  | 0.742 |  |  |  |  |  |  |  |  |  |  |
| SCN1 I have a hard time ... |  |  |  | 0.676 |  |  |  |  |  |  |  |  |  |
| SCN2 I say inappropriate … |  |  |  | 0.733 |  |  |  |  |  |  |  |  |  |
| SCN3 I do certain things ... |  |  |  | 0.771 |  |  |  |  |  |  |  |  |  |
| SCN4 Pleasure and fun … |  |  |  | 0.591 |  |  |  |  |  |  |  |  |  |
| SCN5 I have trouble … |  |  |  | 0.710 |  |  |  |  |  |  |  |  |  |
| SCN6 Sometimes I can’t stop … |  |  |  | 0.776 |  |  |  |  |  |  |  |  |  |

Note. Continued on next page.

Table S4 (Continued)

|  | PRE | COG | CONS | S-C | SS | | SI | | RI | | SIM | UNQ | ATA |
| --- | --- | --- | --- | --- | --- | --- | --- | --- | --- | --- | --- | --- | --- |
|  |  |  |  |  | NOV | INT | NORM | INFO | FIN | ETH |  |  |  |
| SSN1 I would like to … |  |  |  |  | 0.682 |  |  |  |  |  |  |  |  |
| SSN2 I would have … |  |  |  |  | 0.784 |  |  |  |  |  |  |  |  |
| SSN3 If it were possible … |  |  |  |  | 0.726 |  |  |  |  |  |  |  |  |
| SSI1 If I were to go to an ... |  |  |  |  |  | 0.571 |  |  |  |  |  |  |  |
| SSI2 In general, I work better … |  |  |  |  |  | 0.710 |  |  |  |  |  |  |  |
| SSI3 I like the feeling of ... |  |  |  |  |  | 0.729 |  |  |  |  |  |  |  |
| SIN1 When buying products … |  |  |  |  |  |  | 0.863 |  |  |  |  |  |  |
| SIN2 If other people can see me … |  |  |  |  |  |  | 0.901 |  |  |  |  |  |  |
| SIN3 I achieve a sense of ... |  |  |  |  |  |  | 0.842 |  |  |  |  |  |  |
| SII1 If I have little experience … |  |  |  |  |  |  |  | 0.608 |  |  |  |  |  |
| SII2 I often consult … |  |  |  |  |  |  |  | 0.829 |  |  |  |  |  |
| SII3 I frequently gather … |  |  |  |  |  |  |  | 0.810 |  |  |  |  |  |
| RIF1 ...horse races. |  |  |  |  |  |  |  |  | 0.875 |  |  |  |  |
| RIF2 … high-stake poker game. |  |  |  |  |  |  |  |  | 0.895 |  |  |  |  |
| RIF3 … a sporting event. |  |  |  |  |  |  |  |  | 0.879 |  |  |  |  |
| RIE1 Passing off somebody … |  |  |  |  |  |  |  |  |  | 0.766 |  |  |  |
| RIE2 Revealing a … |  |  |  |  |  |  |  |  |  | 0.668 |  |  |  |
| RIE3 Leaving your … |  |  |  |  |  |  |  |  |  | 0.771 |  |  |  |
| SIM1 When a product I own ...* |  |  |  |  |  |  |  |  |  |  | 0.843 |  |  |
| SIM2 I often try to avoid ...* |  |  |  |  |  |  |  |  |  |  | 0.887 |  |  |
| SIM3 As a rule, I dislike ...* |  |  |  |  |  |  |  |  |  |  | 0.803 |  |  |
| SIM4 The more commonplace ...* |  |  |  |  |  |  |  |  |  |  | 0.799 |  |  |
| UNI1 I often combine … |  |  |  |  |  |  |  |  |  |  |  | 0.727 |  |
| UNI2 I often try to find … |  |  |  |  |  |  |  |  |  |  |  | 0.647 |  |
| UNI3 Having an eye … |  |  |  |  |  |  |  |  |  |  |  | 0.724 |  |
| UNI4 When it comes … |  |  |  |  |  |  |  |  |  |  |  | 0.611 |  |
| ATA1 Advertising is essential. |  |  |  |  |  |  |  |  |  |  |  |  | 0.619 |
| ATA2 In general, advertising … |  |  |  |  |  |  |  |  |  |  |  |  | 0.518 |
| ATA3 Advertising helps … |  |  |  |  |  |  |  |  |  |  |  |  | 0.823 |
| ATA4 Advertising results … |  |  |  |  |  |  |  |  |  |  |  |  | 0.825 |

Note.PRE - Premeditation, COG - Cognition, CONS - Consistency, S-C - Self-control, SS - Sensation Seeking (NOV - Novelty, INT - Intensity), SI - social Influence (NORM - Normative, INFO -Informative), RI - Risk Preferences (FIN - Financial, ETH - Ethical), SIM - Similarity, UNQ - Uniqueness, ATA - Attitude to Advertising
