# Supplementary material for: We will make you like our research: The development of a susceptibility-to-persuasion scale
Source: PLoS One. 2018 Mar 15;13(3):e0194119. doi: 10.1371/journal.pone.0194119 (PMC5854354; doi:10.1371/journal.pone.0194119)
Supplement: S5 Table — (DOCX) [file pone.0194119.s006.docx]

Table S5. Standardised Factor Loadings / Correlations Across Items in StP-IIB Factors (n = 279) in Confirmatory Factor Analysis.

|  | Factor | | | | | | | | | |
| --- | --- | --- | --- | --- | --- | --- | --- | --- | --- | --- |
|  | PRE | COG | CONS | S-C | SS | SI | RI | SIM | UNQ | ATA |
| PRE1 I only act … itself. | 0.830 |  |  |  |  |  |  |  |  |  |
| PRE4 I think that sacrificing … | 0.795 |  |  |  |  |  |  |  |  |  |
| PRE5 I only act .. date. | 0.803 |  |  |  |  |  |  |  |  |  |
| COG1 I would rather do something … |  | 0.811 |  |  |  |  |  |  |  |  |
| COG2 I try to anticipate … |  | 0.821 |  |  |  |  |  |  |  |  |
| COG4 Learning new ways … |  | 0.752 |  |  |  |  |  |  |  |  |
| CON3 The appearance of consistency ... |  |  | 0.742 |  |  |  |  |  |  |  |
| CON4 An important requirement ... |  |  | 0.786 |  |  |  |  |  |  |  |
| CON6 I make an effort to … |  |  | 0.791 |  |  |  |  |  |  |  |
| SCN2 I say inappropriate … |  |  |  | 0.697 |  |  |  |  |  |  |
| SCN3 I do certain things ... |  |  |  | 0.796 |  |  |  |  |  |  |
| SCN6 Sometimes I can’t stop … |  |  |  | 0.782 |  |  |  |  |  |  |
| SSN1 I would like to … |  |  |  |  | 0.701 |  |  |  |  |  |
| SSN2 I would have … |  |  |  |  | 0.754 |  |  |  |  |  |
| SSN3 If it were possible … |  |  |  |  | 0.740 |  |  |  |  |  |
| SIN1 When buying products … |  |  |  |  |  | 0.858 |  |  |  |  |
| SIN2 If other people can see me … |  |  |  |  |  | 0.902 |  |  |  |  |
| SIN3 I achieve a sense of ... |  |  |  |  |  | 0.846 |  |  |  |  |
| RIF1 ...horse races. |  |  |  |  |  |  | 0.862 |  |  |  |
| RIF2 … high-stake poker game. |  |  |  |  |  |  | 0.894 |  |  |  |
| RIF3 … a sporting event. |  |  |  |  |  |  | 0.890 |  |  |  |
| SIM1 When a product I own ...* |  |  |  |  |  |  |  | 0.858 |  |  |
| SIM2 I often try to avoid ...* |  |  |  |  |  |  |  | 0.880 |  |  |
| SIM4 The more commonplace ...* |  |  |  |  |  |  |  | 0.792 |  |  |
| UNI1 I often combine … |  |  |  |  |  |  |  |  | 0.720 |  |
| UNI2 I often try to find … |  |  |  |  |  |  |  |  | 0.664 |  |
| UNI3 Having an eye … |  |  |  |  |  |  |  |  | 0.734 |  |
| ATA1 Advertising is essential. |  |  |  |  |  |  |  |  |  | 0.637 |
| ATA3 Advertising helps … |  |  |  |  |  |  |  |  |  | 0.808 |
| ATA4 Advertising results … |  |  |  |  |  |  |  |  |  | 0.842 |

Note.PRE - Premeditation, COG - Cognition, CONS - Consistency, S-C - Self-control, SS - Sensation Seeking, SI - social Influence, RI - Risk Preferences, SIM - Similarity, UNQ - Uniqueness, ATA - Attitude to Advertising
